# The stability of graphene and boron nitride for III-nitride epitaxy and post-growth exfoliation†

**DOI:** 10.1039/d1sc01642c

**Published:** 2021-05-05

**Authors:** Jeong-Hwan Park, Xu Yang, Jun-Yeob Lee, Mun-Do Park, Si-Young Bae, Markus Pristovsek, Hiroshi Amano, Dong-Seon Lee

**Affiliations:** Department of Electronics, Nagoya University Nagoya 464-8603 Japan; School of Electrical Engineering and Computer Science (EECS), Gwangju Institute of Science and Technology (GIST) Gwangju 61005 Republic of Korea dslee66@gist.ac.kr; Institute of Materials and Systems for Sustainability (IMaSS), Nagoya University Nagoya 464-8601 Japan x_yang@echo.nuee.nagoya-u.ac.jp amano@nuee.nagoya-u.ac.jp; Energy Materials Center, Korea Institute of Ceramic Engineering and Technology (KICET) Jinju 52851 Republic of Korea

## Abstract

A challenging approach, but one providing a key solution to material growth, remote epitaxy (RE)—a novel concept related to van der Waals epitaxy (vdWE)—requires the stability of a two-dimensional (2-D) material. However, when graphene, a representative 2-D material, is present on substrates that have a nitrogen atom, graphene loss occurs. Although this phenomenon has remained a hurdle for over a decade, restricting the advantages of applying graphene in the growth of III-nitride materials, few previous studies have been conducted. Here, we report the stability of graphene on substrates containing oxygen or nitrogen atoms. Graphene has been observed on highly decomposed Al_2_O_3_; however, graphene loss occurred on decomposed AlN at temperatures over 1300 °C. To overcome graphene loss, we investigated 2-D hexagonal boron nitride (h-BN) as an alternative. Unlike graphene on AlN, it was confirmed that h-BN on AlN was intact after the same high-temperature process. Moreover, the overgrown AlN layers on both h-BN/AlN and h-BN/Al_2_O_3_ could be successfully exfoliated, which indicates that 2-D h-BN survived after AlN growth and underlines its availability for the vdWE/RE of III-nitrides with further mechanical transfer. By enhancing the stability of the 2-D material on the substrate, our study provides insights into the realization of a novel epitaxy concept.

## Abstract 2

A challenging approach, but one providing a key solution to material growth, remote epitaxy (RE)—a novel concept related to van der Waals epitaxy (vdWE)—requires the stability of a two-dimensional (2-D) material.

## Graphical abstract

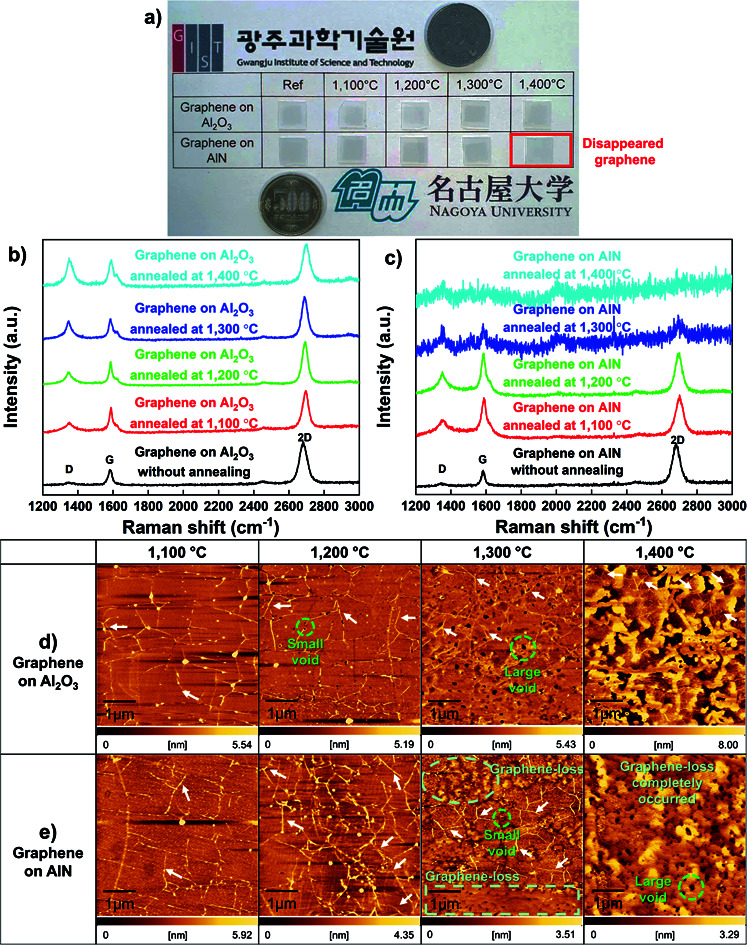

## Introduction

Placing a two-dimensional (2-D) material at the interface between a grown material and a substrate opens novel opportunities for semiconductor devices. The epitaxy caused by a 2-D material, termed van der Waals epitaxy (vdWE), reduces the dislocation density of the grown material, which strongly affects the device properties, and also allows exfoliation of the grown material from the substrate owing to the weak chemical bond.^1–4^ The advantages of vdWE have been confirmed using various structures, such as gallium nitride (GaN) or aluminium nitride (AlN) growth on graphene/silicon carbide (SiC),^5^ graphene/silicon dioxide (SiO_2_),^6–9^ graphene/silicon (Si),^10–12^ graphene/sapphire (Al_2_O_3_),^13–18^ and hexagonal boron nitride (h-BN)/Al_2_O_3_.^19,20^ However, the remaining dislocation density on vdWE is still a hurdle to achieving high-quality semiconductors. Recently, the results of applying graphene at an interface similar to that of the homo-epitaxy have been reported using gallium arsenide (GaAs)/graphene/GaAs,^21^ zinc oxide (ZnO)/graphene/ZnO,^22^ and strontium titanate (SrTiO_3_)/graphene/SrTiO_3_;^23^ the structures of these materials are a result of the interaction between the grown material and the substrate. This structure (*i.e.*, the 2-D material located between the same material) is called remote-epitaxy (RE); specifically, the exfoliation of the overgrown material occurs as a result of the weak interaction at the interface, and the dislocation-free structure is caused by graphene transparency.^24^ In conventional GaN growth on SiC, Si (111), and Al_2_O_3_, the high dislocation density results in a critical problem owing to lattice mismatch, which typically deteriorates the device quality. Additionally, the strong chemical bond between the epitaxially grown GaN and conventional substrates limits the possibility of using this material in various fields of application. RE can pave the way to overcoming the problems of conventional GaN growth; however, the application of RE has been very challenging for materials containing a nitrogen (N) atom, such as the structure of III-nitride/graphene/III-nitride. It has been almost a decade since the research on GaN growth on graphene was reported.^6^ Meanwhile, in our previous study, it was highlighted that if a substrate supporting graphene has a N atom (*e.g.*, GaN), graphene-loss occurs under a conventional growth environment, unlike other reported N-free substrates.^25,26^ This graphene-loss problem remains unsolved and must be overcome in order to realize RE on III-nitride materials.

As graphene has an extremely low surface energy, it is difficult to directly grow III-nitride materials upon it. To allow III-nitride growth on graphene, a potential approach is to generate sp^3^ bonds on graphene. Recently, N-doped graphene with carbon (C)–N bonding has been demonstrated by annealing in ambient ammonia (NH_3_)^7,27^ and using N_2_ plasma treatment.^13,16^ Feng *et al.* have successfully created a GaN/graphene/SiO_2_ structure using ambient NH_3_ annealing on graphene.^7^ Chen *et al.* have also shown UV-LED growth on graphene/Al_2_O_3_ using N_2_ plasma treatment before growth.^16^ These results confirm that N-doped graphene on a substrate containing an oxygen (O) atom is a promising approach to enabling vdWE. Moreover, Jeong *et al.* have recently shown that graphene is stable on a substate containing an O atom (*i.e.*, Al_2_O_3_) and that the substrate can be reused for the remote heteroepitaxy of a GaN microrod light emitting diode (LED).^18^ Meanwhile, regarding the RE structure of III-nitride/graphene/III-nitride, the stability of the 2-D material on the decomposed III-nitride substrate warrants investigation because the growth temperature of III-nitride is higher than its decomposition temperature,^28^ as shown in Fig. S1a,† which means that there is a possibility that column III atoms and N atoms generated from the decomposition of the III-nitride substrate affect graphene. Despite this, the influence of the decomposition of the III-nitride substrate on graphene is still unclear and is yet to be fully investigated.

Here, we report the results of an investigation into the cause of graphene-loss and suggest an alternative 2-D material for vdWE or RE of N-containing III-nitrides. We investigated the stability of two different 2-D materials, graphene and h-BN, on substrates containing either N or O as shown in Fig. S1b† (a more detailed process in reported in the Experimental section). To accurately demonstrate the influence of the N atom, while excluding the influence of the metal generated by the decomposed substrate, Al_2_O_3_ and AlN were used as substrates to support the graphene. Graphene could still be observed on even the highly decomposed Al_2_O_3_ at over 1200 °C, but disappeared on the decomposed AlN at over 1300 °C. Conversely, h-BN grown on AlN was intact after annealing at 1400 °C. We further demonstrated that the overgrown AlN layers on both h-BN/AlN and h-BN/Al_2_O_3_ could be successfully exfoliated, which indicates h-BN is robust during metal–organic chemical vapor deposition (MOCVD) of III-nitrides and could be used as an alternative for vdWE.

## Results and discussion

**Fig. 1:**
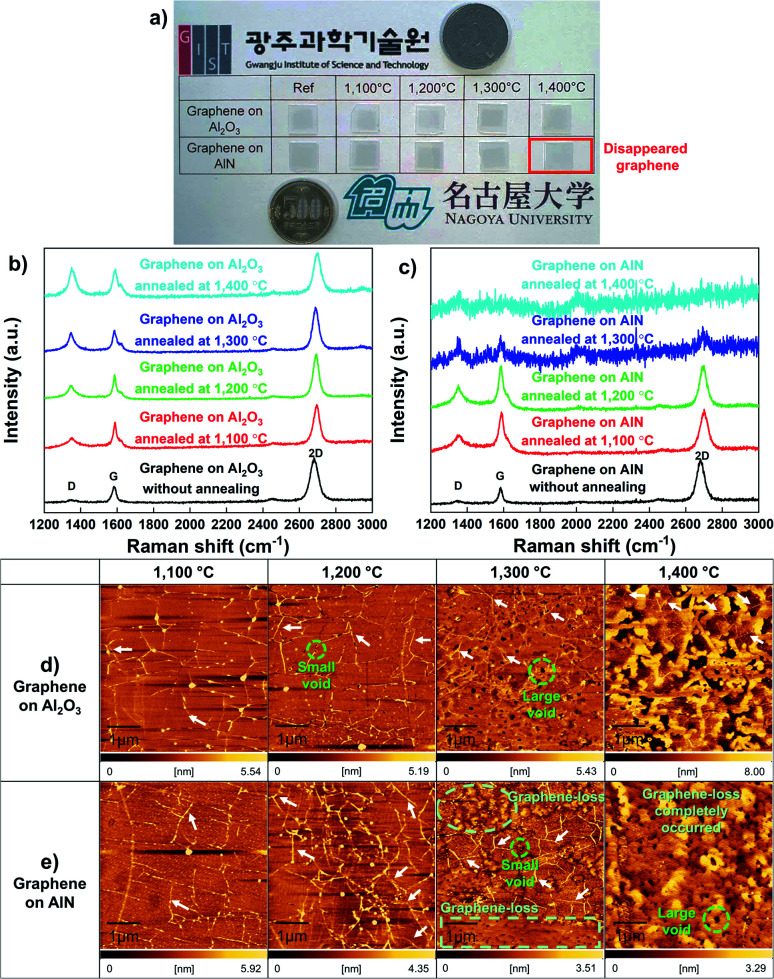
(a) Photographs of annealed graphene on Al_2_O_3_ and AlN at each temperature. The black sheet is graphene and can be distinguished by the naked eye. At 1400 °C, graphene-loss only occurred completely on AlN. Raman spectra of (b) graphene on Al_2_O_3_ and (c) graphene on AlN annealed at each temperature. AFM images of annealed graphene at each temperature (d) on Al_2_O_3_ and (e) on AlN. The white arrows indicate graphene wrinkles.

To investigate the stability of graphene on a substrate at high temperatures, graphene was transferred to Al_2_O_3_ and AlN and annealed over 1100 °C for 10 min in ambient hydrogen (H_2_) by MOCVD. Previous studies on the influence of H_2_ on graphene have shown that H_2_ passivates broken points of graphene accompanied by dangling bonds, which could help move a large atom, such as gallium,^29^ aluminum,^30^ and indium^31^ under graphene; however, the aforementioned studies also show that H_2_ does not result in graphene-loss. Therefore, we used H_2_ to obtain substrate decomposition at a relatively low temperature rather than ambient inert gas. The photograph of the annealing results shown in Fig. 1a reveals that the presence of graphene can be distinguished using the naked eye. Graphene on Al_2_O_3_ annealed at each temperature is observed as a black sheet; however, there is no trace of graphene on AlN annealed at 1400 °C. Therefore, we investigated why and how the graphene-loss occurs on AlN. Fig. 1b and c shows that the stability of graphene on Al_2_O_3_ and AlN was evaluated using Raman measurements. To analyse the effect of substrate decomposition in detail depending on the temperature, we reveal the fitting data of the D, D′, and 2D peaks at 1300–1700 cm^−1^ in Fig. S2.† Additionally, to enhance the measurement reliability, several measurement points are shown in Fig. S3 and S4.† Regardless of the annealing temperature, Fig. 1b shows graphene-related D, G, and 2D peaks were confirmed on Al_2_O_3_ close to 1350, 1580 and 2700 cm^−1^, respectively. With an increase in the annealing temperature, Table 1 shows that the *I*_D_/*I*_G_ also increases. These defect points are closely related to the decomposition of Al_2_O_3_, the critical temperature point of which is approximately 1200 °C in ambient H_2_.^32^ Over 1200 °C, the aluminium (Al)–O bond is broken and Al, and O atoms are generated. This O atoms create the D and D′ peaks of graphene, which are similar to those of the O_2_ plasma-treated graphene.^33,34^ It is also accurately demonstrated that Al generated by Al_2_O_3_ decomposition does not lead to graphene-loss because graphene is observed across the whole surface of all samples over 1200 °C. From the Raman data alone, as shown in Fig. 1b, we can assume that the decomposed Al_2_O_3_ substrate affects the D and D′ peak of graphene, but it does not lead to graphene-loss. However, in the case of the decomposed AlN containing the same atom, Al, even though it has a much higher decomposition temperature than Al_2_O_3_,^35^ graphene completely disappears at 1400 °C. Regarding the result of annealing at 1300 °C, graphene only partially remained on AlN, as shown in the scanning electron microscopy (SEM) images shown in Fig. S5.† When the temperature reached 1400 °C, graphene clearly disappeared, and G and 2D Raman peaks were not observed; however, one peak was observed close to 2000 cm^−1^, which was attributed to the AlN peaks by the reference data shown in Fig. S6.† Atomic force microscopy (AFM) measurements in Fig. 1d and e also agree well with the results obtained from the Raman studies. Although the Al_2_O_3_ surface gradually became rough as the annealing temperature increased, graphene wrinkles were still observed, even at 1400 °C. In contrast, on AlN at 1300 °C, the starting temperature of AlN decomposition, graphene wrinkles and the decomposed surface were simultaneously observed, which means that graphene could disappear owing to the decomposition of AlN containing a N atom. The voids were commonly observed on Al_2_O_3_ and AlN from 1200 and 1300 °C, respectively, and the void size increased along with the annealing temperature, which implies that voids were generated by substrate decomposition.^32,35,36^ Moreover, as shown in Fig. S7,† the depth of the voids was also increased with elevated annealing temperature after the onset of AlN decomposition. We further investigated the stability of graphene on AlN and Al_2_O_3_ at 1400 °C in ambient inert gas (*i.e.*, ambient N_2_) as shown in Fig. S8.† Unlike in ambient H_2_, the decomposition of AlN was negligible in ambient inert gas, even though the temperature reached 1400 °C.^35^ As a result, graphene was observed on the AlN layer after the annealing process. Moreover, using Raman spectroscopy we also confirmed that graphene on Al_2_O_3_ is partially oxidized into graphene oxide after the same annealing process. This observation is consistent with a previous report by Akiyama *et al.*, in which the authors found that marked decomposition of Al_2_O_3_ starts at around 1450 °C.^32^ It is therefore reasonable to suggest that the graphene oxide observed in this study resulted from the O atom arising from the slightly decomposed Al_2_O_3_ owing to the reduced 2D peak intensity, unlike graphene on AlN.

**Table 1:** *I*_D_/*I*_G_ and 
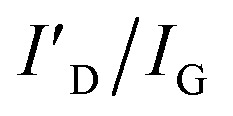
 ratio of graphene on Al_2_O_3_ and AlN depending on the annealing temperature. The calculated values are averages from Fig. S2–S4. For graphene on AlN annealed at 1300 °C, the values of *I*_D_/*I*_G_ and 
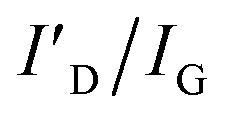
 are calculated from P2, and P3 in Fig. S4c

Annealing temperature	Graphene on Al_2_O_3_		Graphene on AlN	
	*I*_D_/*I*_G_	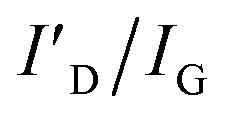	*I*_D_/*I*_G_	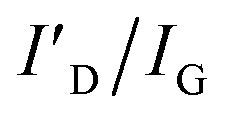
Without annealing	0.248	N.A.	0.279	N.A.
1100 °C	0.479	0.376	0.535	0.444
1200 °C	0.578	0.335	0.695	0.482
1300 °C	0.710	0.370	0.858	0.514
1400 °C	0.987	0.505	N.A.	N.A.

**Fig. 2:**
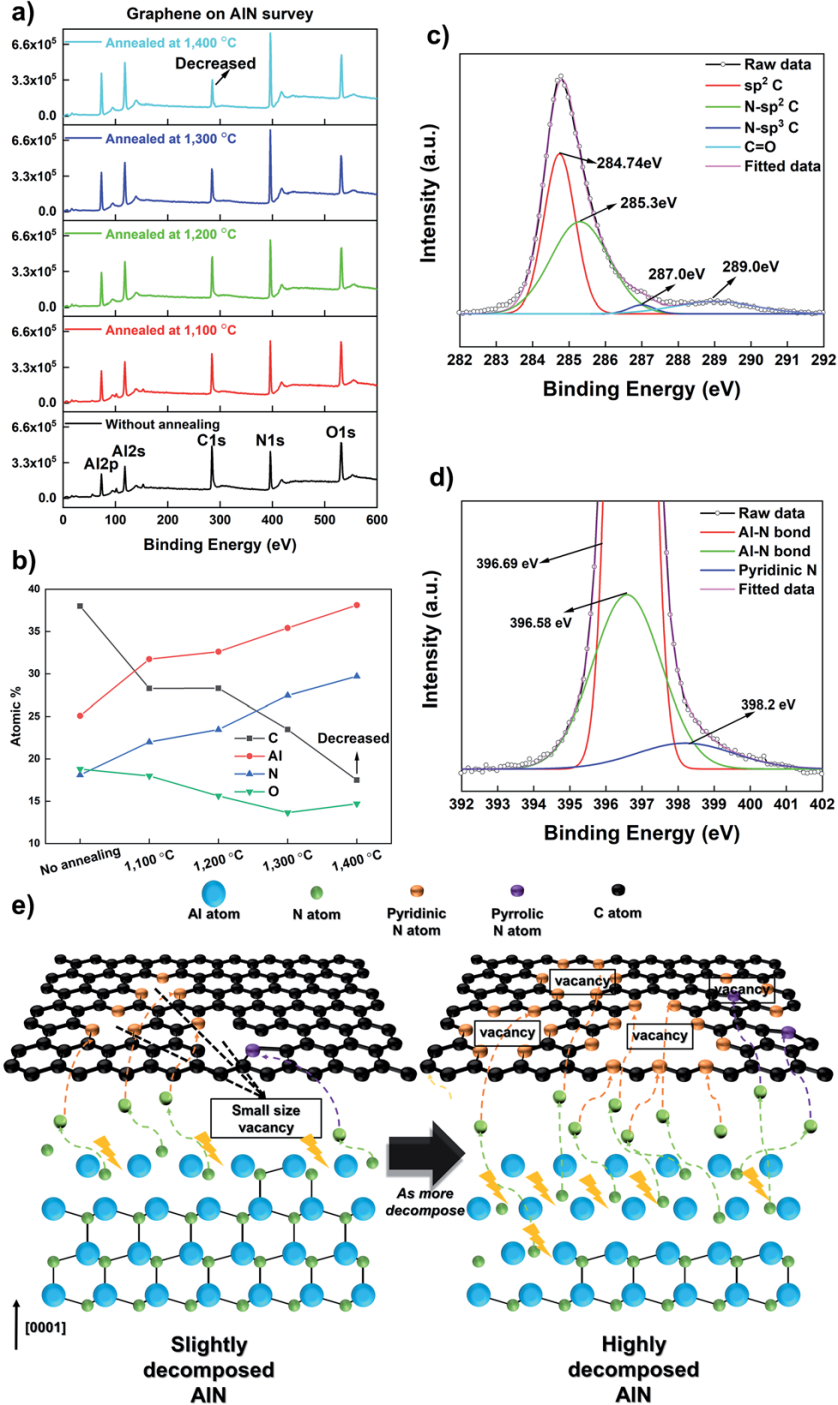
The influence of the N atom generated from substrate decomposition on graphene. (a) XPS spectra of annealed graphene on AlN showing the Al 2p, Al 2s, C 1s, N 1s, and O 1s peaks. (b) The total atomic percentage values of annealed graphene/AlN at the surface. (c) C 1s and (d) N 1s XPS spectra of the annealed graphene on AlN at 1300 °C revealing the signals of C–N bonding. (e) A schematic diagram of the graphene structure affected by the N atom depending on the AlN decomposition.

When the annealing temperature reached 1400 °C, the surface was fully decomposed, and graphene completely disappeared on AlN. It is important to understand why and how graphene-loss occurs on the substrate containing an N atom. We speculate that graphene-loss is closely related to the highly N-doped graphene structure. Barbier *et al.* have recently reported that N-plasma could result in N incorporation in the graphene lattice. Moreover, they demonstrated decreasing X-ray photoelectron spectroscopy (XPS) intensities of C 1s after graphene was exposed to N-plasma.^9^Fig. 2 shows that this N influence agrees well with the results obtained for the decomposition of AlN containing the N atom. Fig. 2a shows the XPS survey for graphene on AlN, along with the annealing temperature depicting the range of the binding energy from 0 to 600 eV. When the annealing temperature increases, the intensity of C 1s decreases, which is supported by the atomic percentage at the surface, as shown in Fig. 2b. Prior to annealing, the ratio of C was approximately 39.16%; however, when the annealing temperature, which triggers the decomposition of the substrate, reached 1400 °C, the ratio of C rapidly decreased to 18.28%. Fig. S9† shows the XPS data of the annealed graphene on Al_2_O_3_, demonstrating that the atomic percentage of C is relatively constant, which clearly differs from that in Fig. 2b. The atomic C percentage of bare AlN is similar to that of the annealed graphene on AlN at 1400 °C, which is 15.24% (not shown here). Following the influence of the N atom on graphene, we fitted the C 1s and N 1s spectra of annealed graphene on AlN at 1300 °C, as shown in Fig. 2c and d, in which graphene was observed by Raman spectroscopy (Fig. S4†) with increased *I*_D_/*I*_G_ and 
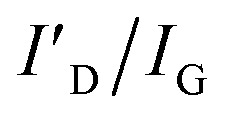
 and is thus very likely to be doped with N. Many reports showed the binding energy of the C–N bonds by measuring XPS. The N-sp^2^ C, N-sp^3^ C, and C=O peak positions are mainly reported at 284.9–285.9, 286.5–287.5, and 288.9–289 eV respectively.^16,37–45^ As shown in Fig. 2c, the C 1s of graphene on AlN annealed at 1300 °C reveals four peak points located at 284.74, 285.3, 287, and 289 eV; these peaks could be attributed to sp^2^ C, N-sp^2^ C, N-sp^3^ C, and C=O respectively. Meanwhile, Ryu *et al.* demonstrated that the decomposition of GaN generates C–N bonds in graphene oxide, but also incorporates N atoms into graphene oxide,^46^ which could occur during the annealing of graphene on AlN over 1300 °C in this work. In general, the N 1s of N-doped graphene is characterized by three peak positions, these are, pyridinic-N (398–399 eV), pyrrolic-N (400–401 eV), and graphitic-N (401–402 eV).^7,16,37–45,47^ Recently, Chen *et al.* demonstrated a AlN/graphene/sapphire structure using N-doped graphene.^48^ They observed N-doped graphene with AlN and showed sp^2^ C–N, and Al–N bonds in the N 1s scan using XPS, although N-doped graphene was covered by AlN. Similar findings were also observed in the N 1s spectrum, as shown in Fig. 2d in this work. The peak near 396.6 eV indicates the Al–N binding energy attributed to AlN.^49^ Moreover, the peak expected for pyridinic-N was observed at 398.2 eV, which implies that the N atom could be incorporated in the graphene lattice. A schematic diagram of the graphene structure affected by the N atom is shown in Fig. 2e. It has been reported that the graphene honeycomb structure is disrupted when doped by the N atom and also vacancy points and holes are generated in graphene accompanied by pyridinic and pyrrolic N, which leads to the loss of C.^37,45,47,50^ Additionally, with an increase in the N-doping concentration, the Raman peaks of graphene gradually fade.^37,45,47^ Compared with the reported N-doped graphene model based on our results, the N atom generated by the decomposed III-nitride substrates could also produce the N-doped graphene, which creates many vacancy areas with a loss of C and, thus the elimination of graphene. The O atom creates defect points on the graphene as sp^3^ bonding and changes graphene to graphene oxide;^51^ however, the influence of the O atom on the graphene-loss is not critical as comparing to that of the N atom, as shown in Fig. 1 and S9.†

**Fig. 3:**
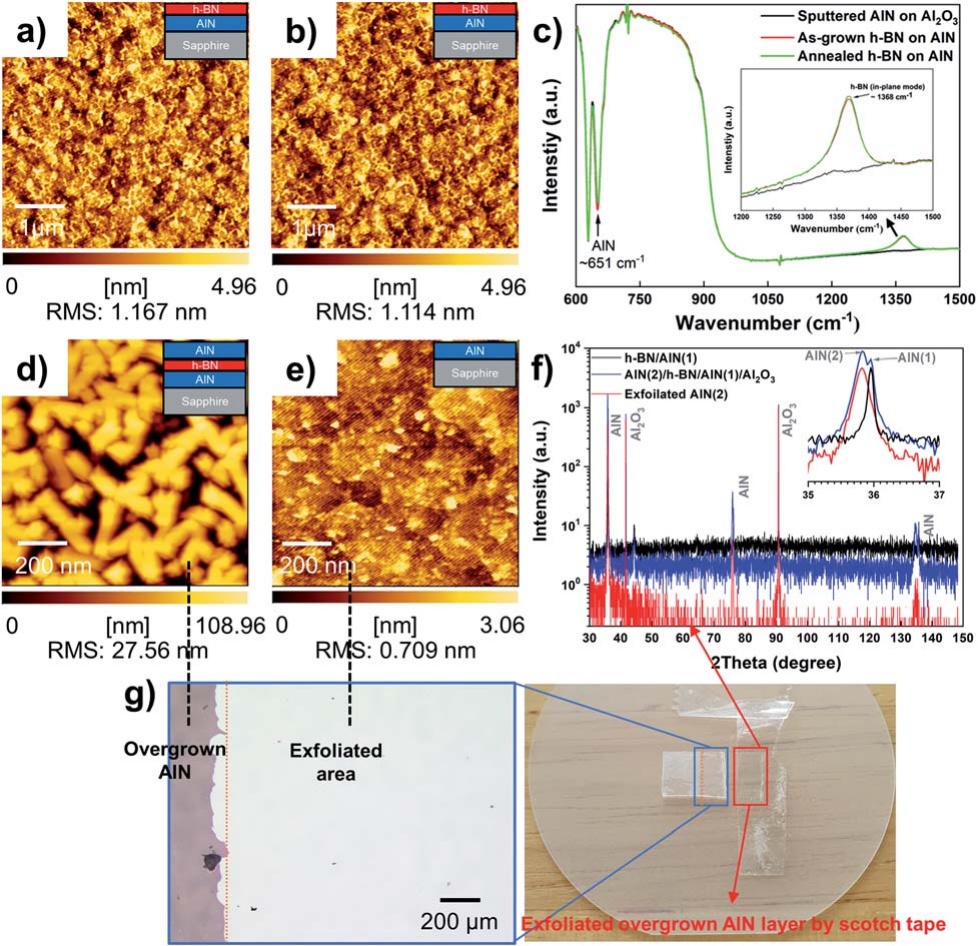
(a) 5 nm-thick h-BN layers grown on AlN at 1380 °C. (b) Annealed h-BN on AlN at 1400 °C for 10 min. There is almost no difference before and after annealing. (c) FTIR spectra of the as-grown and annealed h-BN on AlN. This data reveals that h-BN is stable compared with graphene under the same conditions. (d) An overgrown AlN layer on h-BN/AlN at 1300 °C. (e) The surface morphology after the exfoliation of the overgrown AlN layer. (f) XRD 2theta–omega scan of the AlN layer. (g) Optical microscopy and photographic images showing the overgrown AlN film exfoliated from the substrate.

As reported in previous studies, the 2-D h-BN layer serving as a platform for III-nitrides vdWE and/or RE could be another option to overcome the stability issue of graphene on a substrate that contains the N atom discussed above.^19,20^ For comparison, we also examined the stability of the h-BN film on AlN using an identical annealing process to that used for graphene on AlN in this work. The AFM images in Fig. 3a and b show the surface of the approximately 5 nm-thick h-BN film before and after 1400 °C annealing in ambient H_2_ for 10 min. Clearly, there is almost no difference between these two h-BN surfaces, and the root mean square roughness of the surface before and after annealing is 1.167 and 1.114 nm, respectively. Both the surfaces are decorated by surface wrinkles with a few tiny holes arising from insufficiently coalesced h-BN domains. The h-BN samples were further examined using Fourier transform infrared (FTIR) spectroscopy in the reflection mode. Meanwhile, the AlN/sapphire template was also characterized prior to h-BN deposition. As shown in Fig. 3c, in addition to the feature from the sapphire substrate, all samples exhibit a characteristic vibration mode at approximately 651 cm^−1^, which is correlated to the transverse phonon frequency of AlN.^52^ Moreover, both the as-grown h-BN and annealed one exhibited a clear vibration mode at approximately 1368 cm^−1^, which is associated with the in-plane transverse phonon mode of the sp^2^-bonded h-BN.^53^

Specifically, the peak features for these two h-BN samples are almost the same, which indicates that the effect of the high-temperature (1400 °C) annealing process on the as-grown h-BN film on AlN is negligible. Based on these results, it was confirmed that 2-D h-BN is more stable than graphene on a substrate containing nitrogen. To further examine the stability of h-BN for subsequent AlN overgrowth, around 800 nm-thick of AlN was grown on a template of 5 nm-thick h-BN film on AlN/sapphire. Fig. 3d shows AFM images of the grown AlN layer, which was relatively rough, probably owing to incomplete coalescence. The XRD 2theta–omega scan shown in Fig. 3f shows two distinct AlN reflections, a very narrow one at 35.95° from the h-BN/AlN template and a broader one at around 35.82° relating to the AlN overlayer. Before exfoliation, two AlN peaks were observed at 35.95° and 35.82° simultaneously; however, the exfoliated AlN layer only had a single peak at 35.82°, which indicates the crystallization of the overgrown AlN layer has been retained after exfoliation and only the overgrown AlN layer has been successfully exfoliated. We observed that the exfoliated AlN has an Al_2_O_3_ peak because it was measured on the Al_2_O_3_ substrate after the exfoliation process. Furthermore, Fig. 3g reveals that we could exfoliate the AlN layer from the h-BN/AlN template by using scotch tape, which indicates the interaction between the AlN and h-BN layer is weak and the connection to the underlying AlN must be very small. The surface of the sample after exfoliation is shown in Fig. 3e and was quite different from both the initial h-BN surface (Fig. 3a) and the grown AlN surface (Fig. 3d). Furthermore, the surface image is similar to that of the underlying bare AlN layer shown in Fig. S10.† Hence, it is likely that the h-BN layer was mostly exfoliated together with the overgrown AlN layer.

**Fig. 4:**
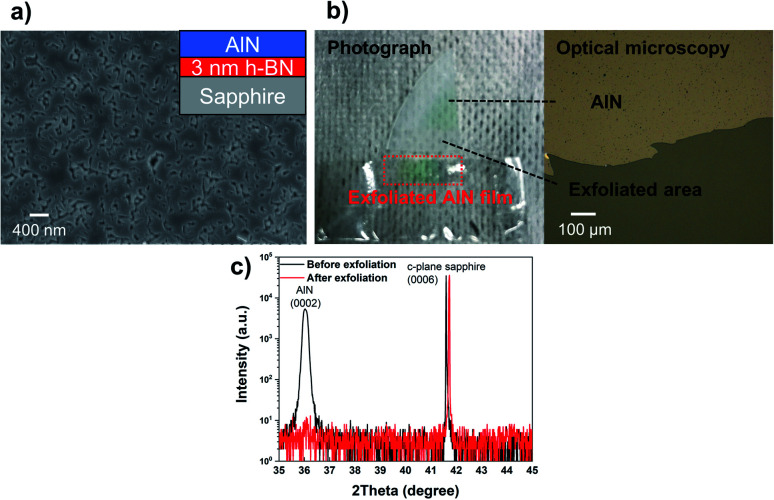
(a) A top-view SEM image of the grown AlN at 1380 °C on h-BN/*c*-plane Al_2_O_3_. (b) Photographic and optical microscopy images showing the exfoliated AlN film caused by the interfacial h-BN layer. (c) XRD 2theta–omega scans of the AlN layer before and after exfoliation.

In addition to the AlN overgrown on h-BN on AlN, we also examined the AlN growth on 3 nm-thick h-BN on sapphire and demonstrated the successful post-growth exfoliation of AlN. Fig. 4a shows the SEM image of the approximately 480 nm-thick AlN grown on h-BN/*c*-plane Al_2_O_3_, in which the formed AlN layer almost coalesced on h-BN/*c*-plane Al_2_O_3_, and this can even be easily exfoliated using scotch tape, as shown in Fig. 4b. However, the same growth on *c*-plane Al_2_O_3_ presents an island-like surface of the AlN layer, as shown in Fig. S11,† and could not be exfoliated. The findings prove that the h-BN layer during AlN growth at 1380 °C survives, resulting in a weak interaction between the epitaxial AlN layer and substrate. This eventually leads to the exfoliation of the AlN epilayer. According to the 2theta–omega scan shown in Fig. 4c, it also indicates that the epitaxially grown AlN overlayer on h-BN is successfully exfoliated. The AlN (0002) peak was observed around 36.0°, whereas it is not detected after the exfoliation process. Once again, this indicates that the peeling-off of the overgrown AlN layer is due to the interfacial h-BN layer.

## Conclusions

In conclusion, we investigated the stability of graphene on substrates containing O or N atoms and demonstrated that graphene was unstable on substrates containing an N atom above its decomposition temperature. In the case of graphene on Al_2_O_3_, graphene was observed even though Al_2_O_3_ was fully decomposed; however, graphene was not observed on the decomposed AlN, which means that the N atom generated by substrate decomposition not only causes graphene loss, but also limits the application of graphene in novel epitaxy concepts. Unlike graphene on AlN, layered h-BN grown on AlN exhibited good stability and did not show significant differences in surface features before and after the annealing process. Furthermore, it was also observed that the grown AlN layers on h-BN/AlN and h-BN/Al_2_O_3_ could be easily exfoliated. All of these results prove that h-BN is stable on the AlN surface and survives during the high-temperature AlN overgrowth process. Therefore, it is necessary to use a substrate that can avoid graphene loss and to select appropriate 2-D materials, such as h-BN, that are sufficiently robust for the realization of a novel epitaxy concept for III-nitrides.

## Author contributions

H. Amano and D.-S. Lee directed and funded this work. J.-H. Park and X. Yang conceived and wrote the manuscript. J.-H. Park, X. Yang, J.-Y. Lee, M.-D. Park, and M. Pristovsek conducted experiments. J.-H. Park, X. Yang, J.-Y. Lee, M.-D. Park, S.-Y. Bae, and M. Pristovsek analysed data. All authors discussed the results and commented on the manuscript.

## Conflicts of interest

There are no conflicts to declare.

## Supplementary Material

SC-012-D1SC01642C-s001

## References

[cit1] Koma A. (1992). Thin Solid Films.

[cit2] Utama M. I. B., Zhang Q., Zhang J., Yuan Y., Belarre F. J., Arbiol J., Xiong Q. (2013). Nanoscale.

[cit3] Yu J., Wang L., Hao Z., Luo Y., Sun C., Wang J., Han Y., Xiong B., Li H. (2019). Adv. Mater..

[cit4] Bae S.-H., Lu K., Han Y., Kim S., Qiao K., Choi C., Nie Y., Kim H., Kum H. S., Chen P., Kong W., Kang B.-S., Kim C., Lee J., Baek Y., Shim J., Park J., Joo M., Muller D. A., Lee K., Kim J. (2020). Nat. Nanotechnol..

[cit5] Kim J., Bayram C., Park H., Cheng C. W., Dimitrakopoulos C., Ott J. A., Reuter K. B., Bedell S. W., Sadana D. K. (2014). Nat. Commun..

[cit6] Chung K., Lee C.-H., Yi G.-C. (2010). Science.

[cit7] Feng Y., Yang X., Zhang Z., Kang D., Zhang J., Liu K., Li X., Shen J., Liu F., Wang T., Ji P., Xu F., Tang N., Yu T., Wang X., Yu D., Ge W., Shen B. (2019). Adv. Funct. Mater..

[cit8] Morassi M., Guan N., Dubrovskii V. G., Berdnikov Y., Barbier C., Mancini L., Largeau L., Babichev A. V., Kumaresan V., Julien F. H., Travers L., Gogneau N., Harmand J.-C., Tchernycheva M. (2020). Cryst. Growth Des..

[cit9] Barbier C., Zhou T., Renaud G., Geaymond O., Fevre P. L., Glas F., Madouri A., Cavanna A., Travers L., Morassi M., Gogneau N., Tchernycheva M., Harmand J.-C., Largeau L. (2020). Cryst. Growth Des..

[cit10] Araki T., Uchimura S., Sakaguchi J., Nanishi Y., Fujishima T., Hsu A., Kim K. K., Palacios T., Pesquera A., Centeno A., Aurutuza A. (2014). Appl. Phys. Express.

[cit11] Zheng Y., Wang W., Li Y., Lan J., Xia Y., Yang Z., He X., Li G. (2019). ACS Appl. Mater. Interfaces.

[cit12] Zulkifli N. A. A., Park K., Min J.-W., Ooi B. S., Zakaria R., Kim J., Tan C. L. (2020). Appl. Phys. Lett..

[cit13] Chen Z., Zhang X., Dou Z., Wei T., Liu Z., Qi Y., Ci H., Wang Y., Li Y., Chang H., Yan J., Yang S., Zhang Y., Wang J., Gao P., Li J., Liu Z. (2018). Adv. Mater..

[cit14] Lundin W. V., Zavarin E. E., Sakharov A. V., Zakheim D. A., Davydov V. Y., Smirnov A. N., Eliseyev I. A., Yagovkina M. A., Brunkov P. N., Lundina E. Y., Markov L. K., Tsatsulnikov A. F. (2018). J. Cryst. Growth.

[cit15] Qi Y., Wang Y., Pang Z., Dou Z., Wei T., Gao P., Zhang S., Xu X., Chang Z., Deng B., Chen S., Chen Z., Ci H., Wang R., Zhao F., Yan J., Yi X., Liu K., Peng H., Liu Z., Tong L., Zhang J., Wei Y., Li J., Liu Z. (2018). J. Am. Chem. Soc..

[cit16] Chen Z., Liu Z., Wei T., Yang S., Dou Z., Wang Y., Ci H., Chang H., Qi Y., Yan J., Wang J., Zhang Y., Gao P., Li J., Liu Z. (2019). Adv. Mater..

[cit17] Jia Y., Ning J., Zhang J., Yan C., Wang B., Zhang Y., Zhu J., Shen X., Dong J., Wang D., Hao Y. (2019). Adv. Optical Mater..

[cit18] Jeong J., Wang Q., Cha J., Jin D. K., Shin D. H., Kwon S., Kang B. K., Jang J. H., Yang W. S., Choi Y. S., Yoo J., Kim J. K., Lee C.-H., Lee S. W., Zakhidov A., Hong S., Kim M. J., Hong Y. J. (2020). Sci. Adv..

[cit19] Kobayashi Y., Kumakura K., Akasaka T., Makimoto T. (2012). Nature.

[cit20] Sundaram S., Li X., Halfaya Y., Ayari T., Patriarche G., Bishop C., Alam S., Gautier S., Voss P. L., Salvestrini J. P., Ougazzaden A. (2019). Adv. Mater. Interfaces..

[cit21] Kim Y., Cruz S. S., Lee K., Alawode B. O., Choi C., Song Y., Johnson J. M., Heidelberger C., Kong W., Choi S., Qiao K., Almansouri I., Fitzgerald E. A., Kong J., Kolpak A. M., Hwang J., Kim J. (2017). Nature.

[cit22] Jeong J., Min K.-A., Shin D. H., Yang W. S., Yoo J., Lee S. W., Hong S., Hong Y. J. (2018). Nanoscale.

[cit23] Kum H. S., Lee H., Kim S., Lindemann S., Kong W., Qiao K., Chen P., Irwin J., Lee J. H., Xie S., Subramanian S., Shim J., Bae S.-H., Choi C., Ranno L., Seo S., Lee S., Bauer J., Li H., Lee K., Robinson J. A., Ross C. A., Schlom D. G., Rzchowski M. S., Eom C. B., Kim J. (2020). Nature.

[cit24] Kong W., Li H., Qiao K., Kim Y., Lee K., Nie Y., Lee D., Osadchy T., Molnar R. J., Gaskill D. K., Myers-ward R. L., Daniels K. M., Zhang Y., Sundram S., Yu Y., Bae S.-H., Rajan S., Shao-horn Y., Cho K., Ougazzaden A., Grossman J. C., Kim J. (2018). Nat. Mater..

[cit25] Park J.-H., Lee J.-Y., Park M.-D., Min J.-H., Lee J.-S., Yang X., Kang S., Kim S.-J., Jeong W.-L., Amano H., Lee D.-S. (2019). Adv. Mater. Interfaces.

[cit26] Lee J.-Y., Min J.-H., Bae S.-Y., Park M.-D., Jeong W.-L., Park J.-H., Kang C.-M., Lee D.-S. (2020). J. Appl. Cryst..

[cit27] Sarau G., Heilmann M., Bashouti M., Latzel M., Tessarek C., Christiansen S. (2017). ACS Appl. Mater. Interfaces.

[cit28] Togashi R., Kamoshita T., Adachi H., Murakami H., Kumagai Y., Koukitu A. (2009). Phys. Status Solidi C.

[cit29] Balushi Z. Y. A., Wang K., Ghosh R. K., Vila R. A., Eichfeld S. M., Caldwell J. D., Qin X., Lin Y.-C., DeSario P. A., Stone G., Subramanian S., Paul D. F., Wallace R. M., Datta S., Redwing J. M., Robinson J. A. (2016). Nat. Mater..

[cit30] Wang W., Zheng Y., Li X., Le Y., Huang L., Yang Z., Zhang X., Li G. (2019). Adv. Mater..

[cit31] Pecz B., Nicotra G., Giannazzo F., Yakimova R., Koos A., Georgieva A. K. (2020). Adv. Mater..

[cit32] Akiyama K., Araki T., Murakami H., Kumagai Y., Koukitu A. (2007). Phys. Status Solidi C.

[cit33] Childers I., Jauregui L. A., Tian J., Chen Y. P. (2011). New J. Phys..

[cit34] Li H., Singh A., Bayram F., Childress A. S., Rao A. M., Koley G. (2019). Nanoscale.

[cit35] Kumagai Y., Akiyama K., Togashi R., Murakami H., Takeuchi M., Kinoshita T., Takada K., Aoyagi Y., Koukitu A. (2007). J. Cryst. Growth.

[cit36] Hagedorn S., Knauer A., Brunner F., Mogilatenko A., Zeimer U., Weyers M. (2017). J. Cryst. Growth.

[cit37] Zhang C., Fu L., Liu N., Liu M., Wang Y., Liu Z. (2011). Adv. Mater..

[cit38] Wang C., Zhou Y., He L., Ng T.-W., Hong G., Wu Q.-H., Gao F., Lee C.-S., Zhang W. (2013). Nanoscale.

[cit39] Xu H., Zhou S., Xiao L., Wang H., Li S., Yuan Q. (2015). J. Mater. Chem. C.

[cit40] Zhang Y., Sun Z., Wang H., Wang Y., Liang M., Xue S. (2015). RSC Adv..

[cit41] Yang T., Qian T., Wang M., Liu J., Zhou J., Sun Z., Chen M., Yan C. (2015). J. Mater. Chem. A.

[cit42] Rybin M., Pereyaslavtsev A., Vasilieva T., Myasnikov V., Sokolov L., Pavlova A., Obraztsova E., Khomich A., Ralchenko V., Obraztsova E. (2016). Carbon.

[cit43] Sakulsermsuk S., Singjai P., Chaiwong C. (2016). Diam. Relat. Mater..

[cit44] Nechiyil D., Vinayan B. P., Ramaprabhu S. (2017). J. Colloid Interf. Sci..

[cit45] Thilawala K. G. N., Kim J. K., Lee J.-M. (2019). J. Alloys Compd..

[cit46] Ryu B. D., Han N., Han M., Chandramohan S., Park Y. J., Ko K. B., Park J. B., Cuong T. V., Hong C.-H. (2014). Mater. Lett..

[cit47] Park S. H., Cha J., Cho M.-H., Kim J. H., Yoo K.-H., Cho S. W., Kim T. G., Kim J. W. (2014). J. Mater. Chem. C.

[cit48] Chen Y., Zang H., Jiang K., Ben J., Zhang S., Shi Z., Jia Y., Lu W., Sun X., Li D. (2020). Appl. Phys. Lett..

[cit49] Alevli M., Ozgit C., Donmez I., Biyikli N. (2012). Phys. Status Solidi A.

[cit50] Zhao W., Hofert O., Gotterbarm K., Zhu J. F., Papp C., Steineruck H. P. (2012). J. Phys. Chem. C.

[cit51] Vervuurt R. H. J., Karasulu B., Verheijen M. A., Kessels W. M. M., Bol A. A. (2017). Chem. Mater..

[cit52] Cheng H., Sun Y., Zhang Z. X., Zhang Y. B., Yuan S., Hing P. (2003). J. Cryst. Growth.

[cit53] Geick R., Perry C. H. (1966). Phys. Rev..

