# Supplementary material for: The stability of graphene and boron nitride for III-nitride epitaxy and post-growth exfoliation
Source: Chem Sci. 2021 May 5;12(22):7713–9. doi: 10.1039/d1sc01642c (PMC8188504; doi:10.1039/d1sc01642c)
Supplement: SC-012-D1SC01642C-s001 [file SC-012-D1SC01642C-s001.pdf]

Electronic Supplementary Material (ESI) for Chemical Science.
This journal is © The Royal Society of Chemistry 2021

Experimental Section

Graphene transfer: The synthesized graphene/copper/graphene (g/Cu/g) received from LG Electronics was transferred by the normal wet transfer method. G/Cu/g was coated by polymethyl methacrylate (PMMA) at 3,000 rpm for 20 s, and Cu foil was etched by $FeCl_3$ for 1 h. After that, PMMA was removed in acetone. Al_2O_3 substrate and AlN template were purchased from Hi-Solar Co., Ltd. AlN template was grown on conventional two-step method by MOCVD. Over 900 °C, AlN buffer was grown on *c*-plane Al_2O_3 and crystalline AlN was grown on buffer layer over 1,250 °C. The growth rate of AlN was 10 μm/h.

H-BN growth: 2-D h-BN layers were epitaxially grown on about 200-nm-thick AlN films on *c*-plane Al_2O_3 substrates by MOCVD (SH4001-HTA, EpiQuest), where AlN films were fabricated by sputtering and high temperature annealing process.[1] As previously reported,[2-3] the growth was conducted using a pulsed-mode method, in which triethylboron and NH_3 as B and N sources, respectively, were alternatingly supplied into the reactor with a nominal V/III ratio of 3,000. The temperature of 1,380 °C and the pressure of 29 Torr were maintained during growth. Finally, around 5 nm layered BN films were grown by tunning the growth pulse as confirmed in our previous report.[4]

Annealing: Graphene and h-BN were annealed at target temperature for 10 min in H_2 ambient (18 SLM) and 200 Torr by MOCVD that was used for h-BN growth experiments in this study. Considering the decomposition temperature, we selected target temperature as 1,100, 1,200, 1,300, and 1,400 °C. The target temperature was raised over 14.5, 16, 17 and 17.5 min respectively.

AlN growth on h-BN/AlN: Around 800 nm AlN layers were grown on h-BN/AlN by two step process. First step is nucleation at 1,150 °C with a V/III ratio of 8,500 followed by crystalline growth at 1,300 °C with a V/III ratio of 325. The process pressure was maintained at 37.5 Torr during growth.

AlN growth on h-BN/sapphire: Around 480 nm AlN layers were grown on *c*-plane Al_2O_3 and h-BN/*c*-plane Al_2O_3 substrates at 1,380 °C with a V/III ratio of 450. The process pressure was maintained at 29 Torr during growth.

Measurement: Graphene samples were analyzed by micro-Raman spectroscopy system equipped with a 514 nm wavelength laser (RENISHAW inVia), SEM (SU-9000, Hitachi), AFM (NanoNavi IIs, SII Nano-Technology), and XPS equipped with an Al-K Alpha

source gun (NEXSA, Thermo Fisher Scientific). The XPS measuring area had an elliptical shape with a long axis of 450 μm and a short axis of 300 μm, and the measurement depth was about 10 nm. Fourier transform infrared (FTIR, FTIR-6100, JASCO) reflectance spectra of the h-BN samples were taken at room temperature in the wavenumber between 600 cm^{-1} and 1500 cm^{-1} with a resolution of 4 cm^{-1}. All spectra were normalized by the reflectance spectrum of an aluminum mirror.

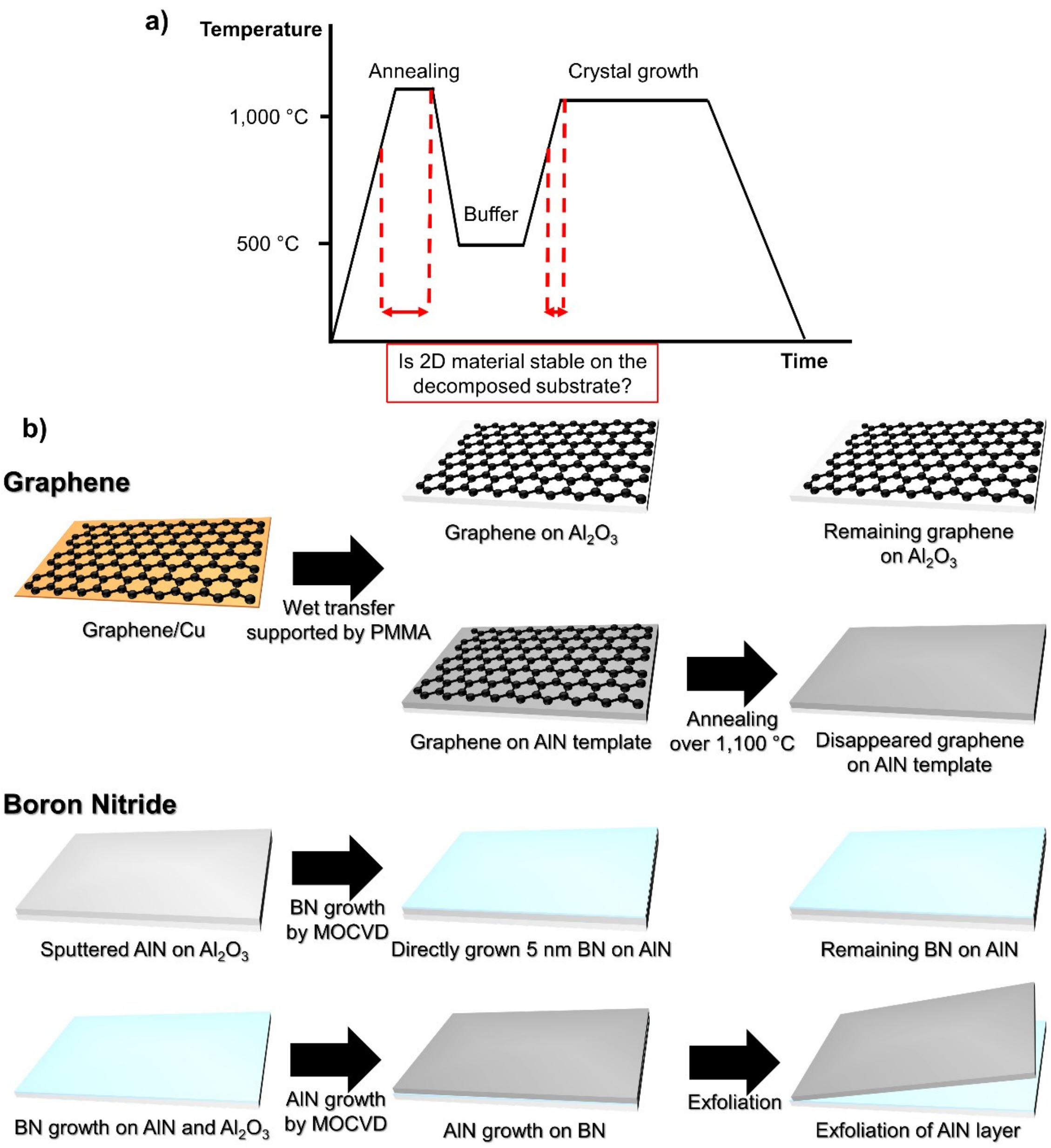

Figure S1. a) Conventional GaN two-step process including a buffer and crystal growth system.[5] The growth temperature of crystal GaN is higher than its decomposition temperature point, which may influence the 2-D material if GaN is used as a substrate to support the 2-D material, e.g., the GaN/graphene/GaN structure. b) Schematic of the sample preparation process. Transferred graphene on Al_2O_3 and AlN template by the wet transfer method and the epitaxially grown BN film on AlN. Each sample was annealed over 1,100 °C by MOCVD. AlN growth on h-BN/AlN and h-BN/Al_2O_3 and its exfoliation caused by the surviving h-BN layer.

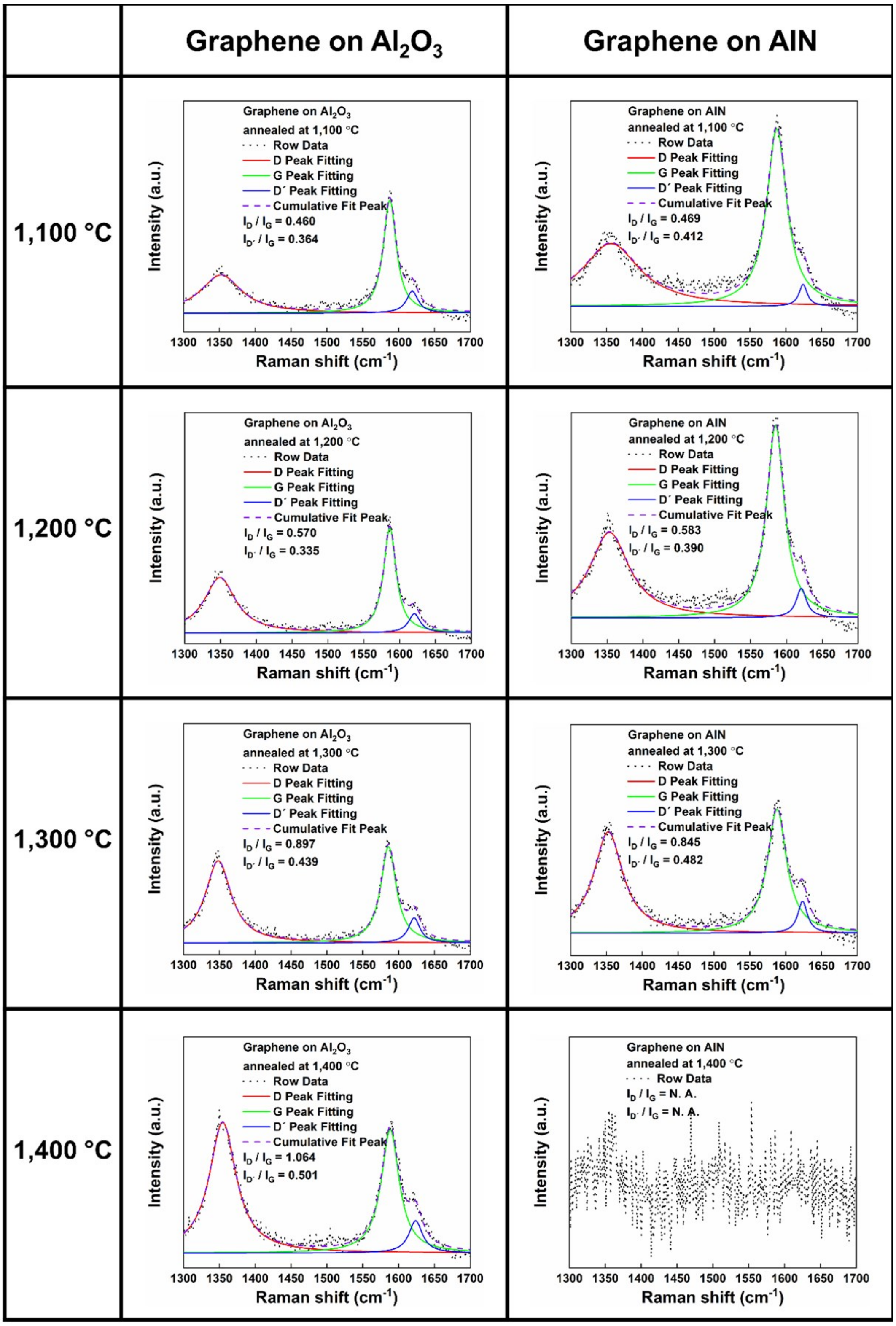

Figure S2. Fitted Raman spectra of graphene on Al_2O_3 and AlN at each temperature. The fitted data exhibit D, D′, and 2D peaks at 1,350, 1,580, and 1,650 cm^{-1}, respectively. Regarding graphene on AlN annealed at 1,300 °C, it is fitted by P2 as shown in Figures S4. There is no peak for graphene on AlN annealed at 1,400 °C.

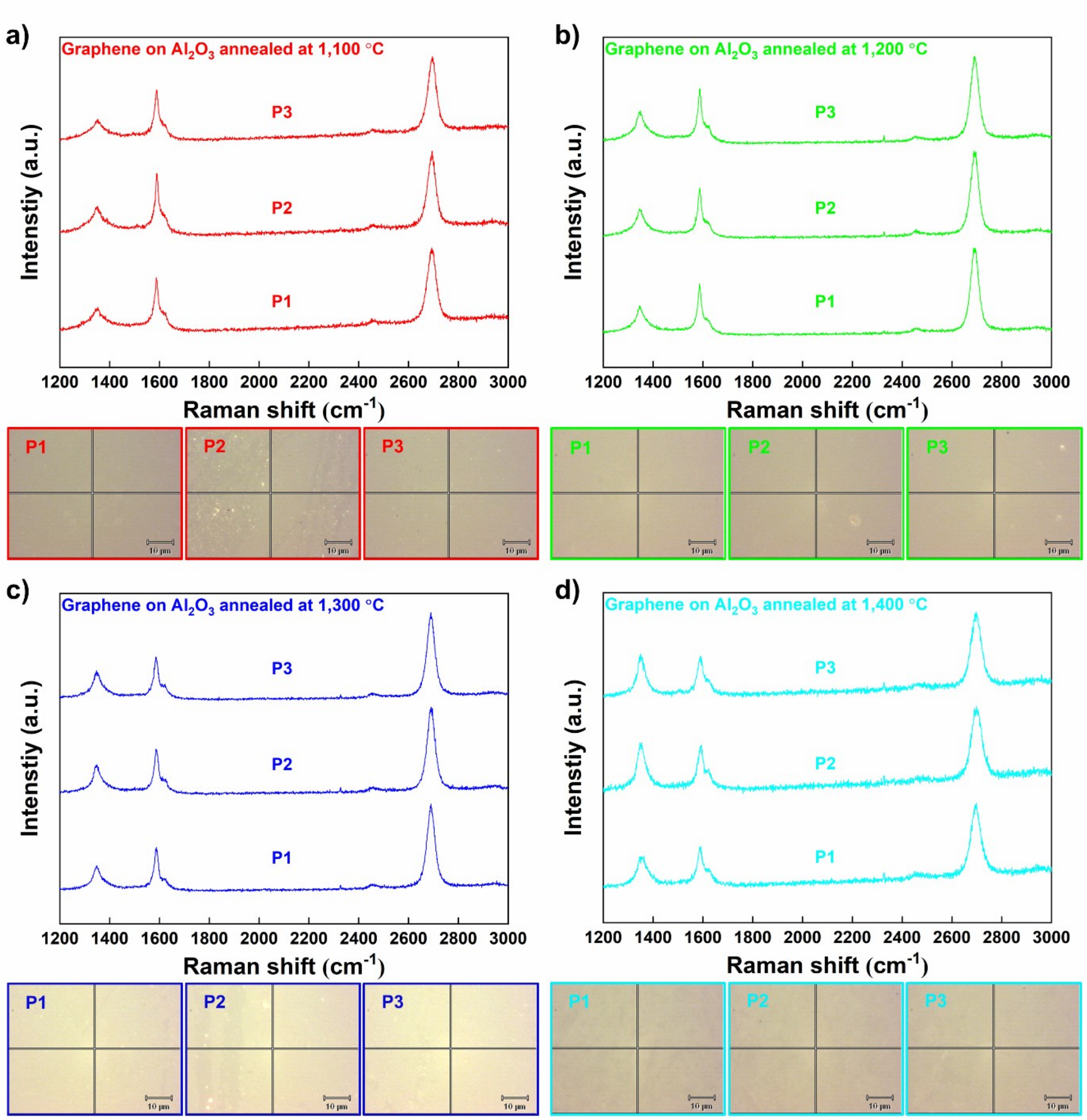

Figure. S3. Raman spectra of graphene on Al_2O_3 annealed at various temperatures for 10 min in H_2 ambient, i.e., at a) 1,100 °C; b) 1,200 °C; c) 1,300 °C; and d) 1,400 °C. Inset images in each figure show the measurement points.

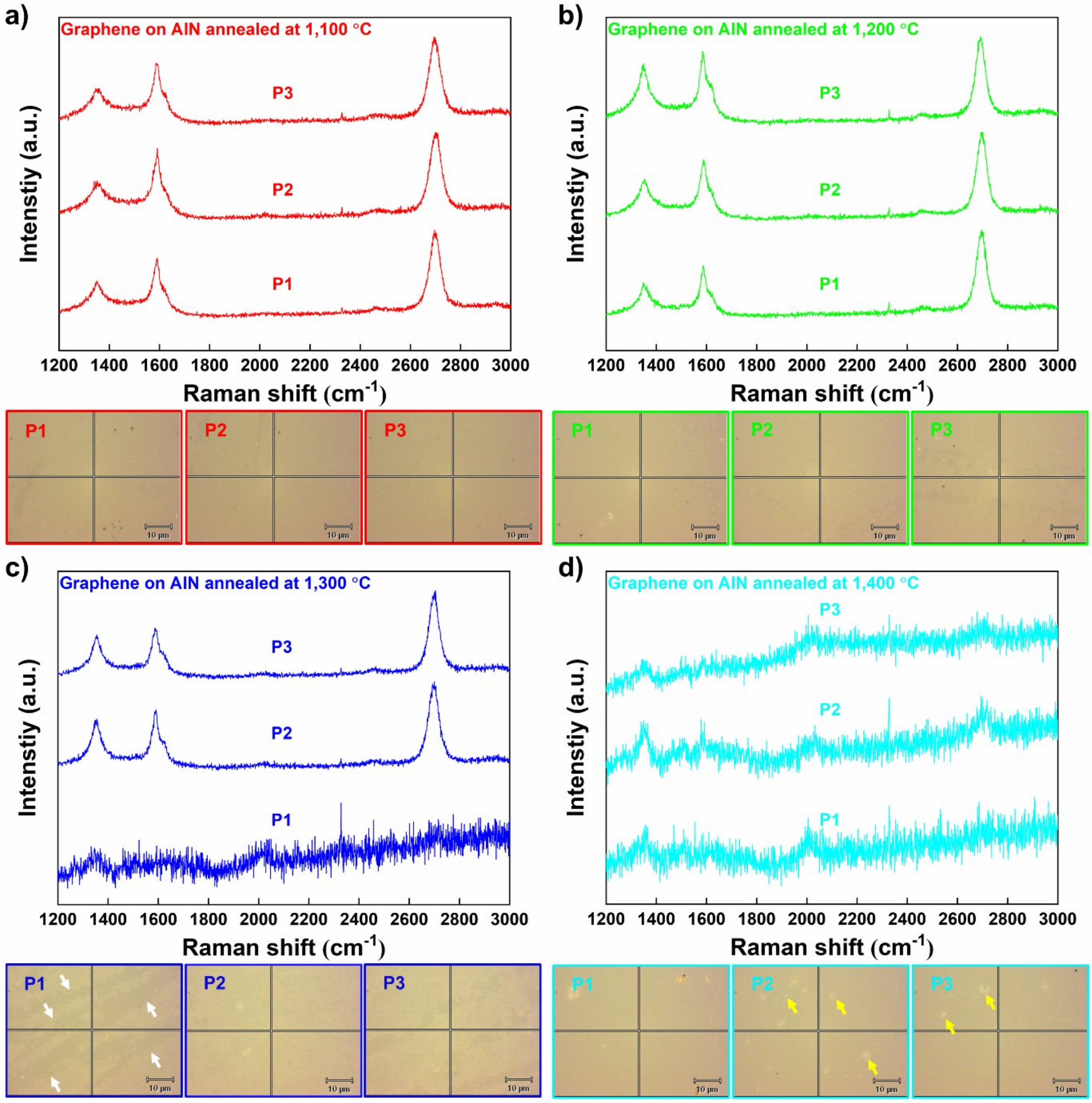

Figure. S4. Raman spectra of graphene on AlN annealed at various temperatures for 10 min in H_2 ambient, i.e., at a) 1,100 °C; b) 1,200 °C; c) 1,300 °C; and d) 1,400 °C. Inset images in each figure show the measurement points. The write and yellow arrows mean graphene-loss and remaining graphene, respectively.

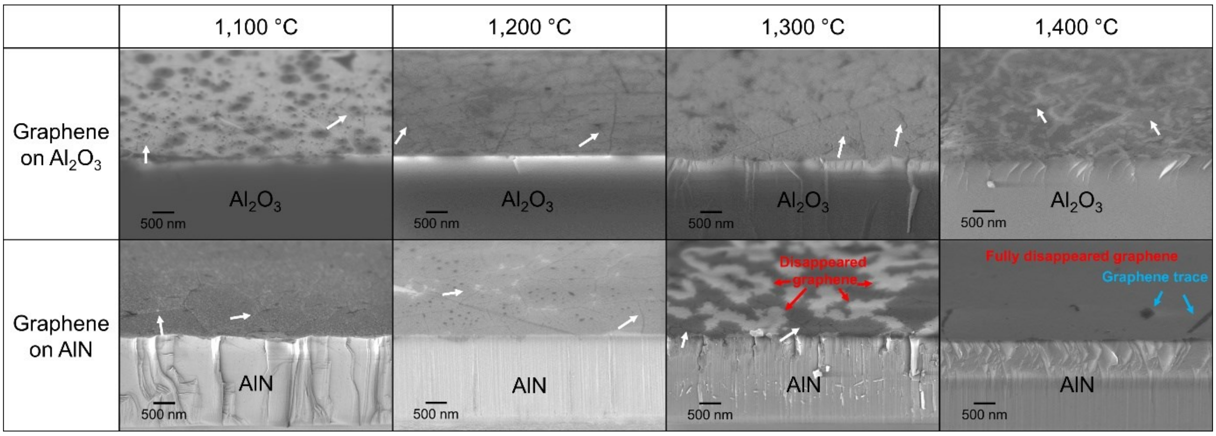

Figure. S5. SEM images of annealed graphene on Al_2O_3 and AlN. White, red, and blue arrows indicate graphene wrinkles, graphene-loss, and graphene trace, respectively.

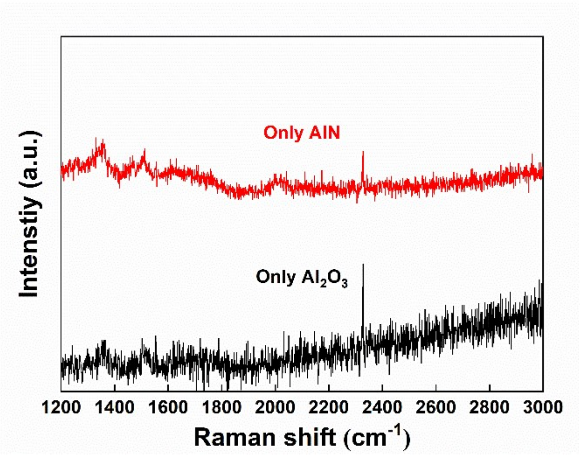

Figure. S6. Raman spectra of bare Al_2O_3 and AlN.

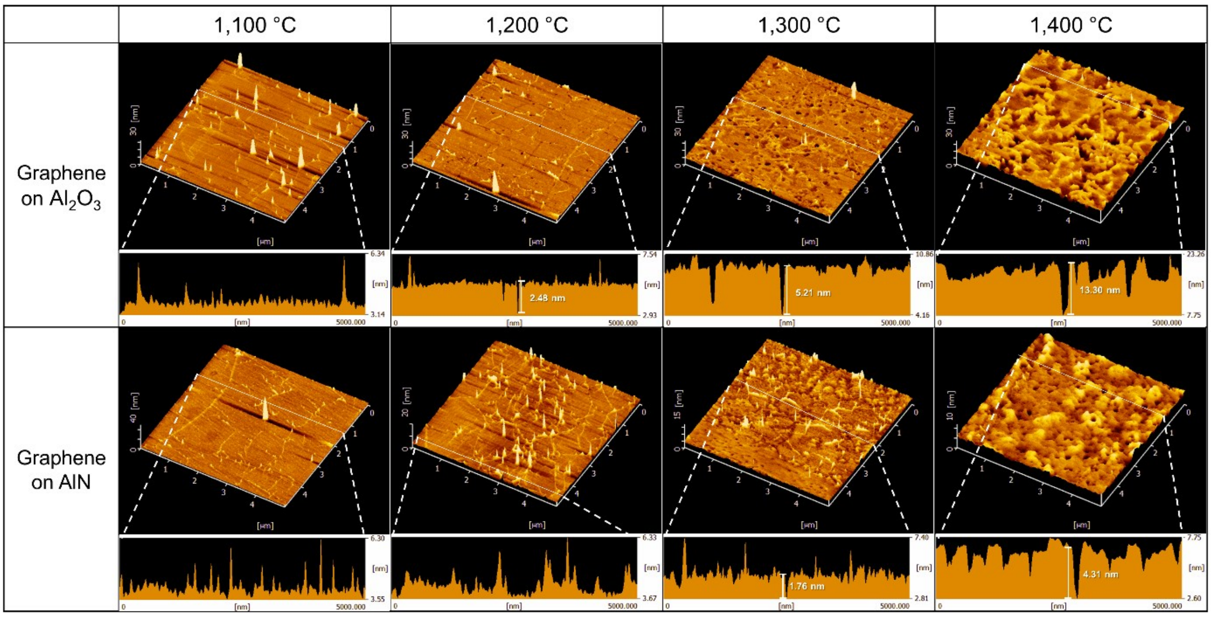

Figure. S7. Tilted AFM image of annealed graphene on each substrate and the void depth.

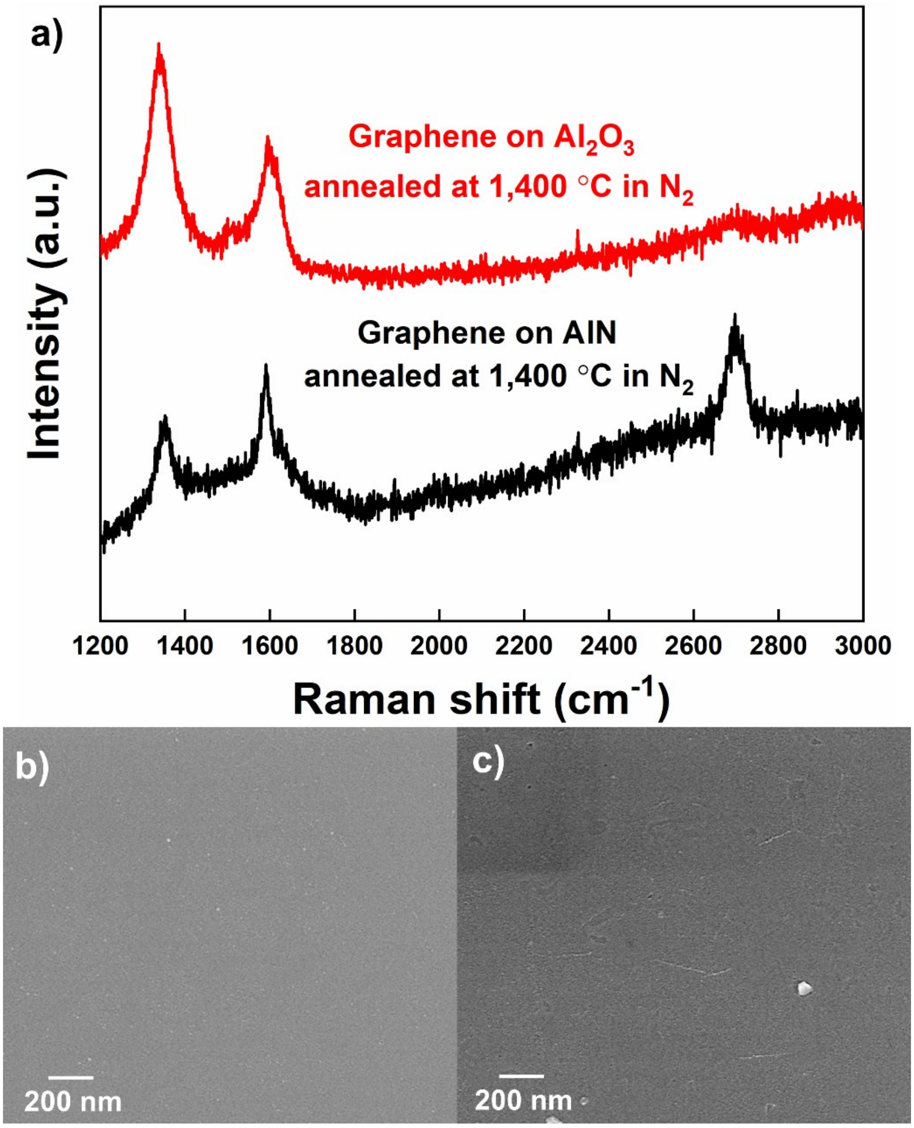

Figure. S8. Annealed graphene at 1,400 °C in N_2 ambient on each substrate. a) Raman spectra of graphene on Al_2O_3 and AlN. Top view SEM image of b) graphene on Al_2O_3 and c) graphene on AlN.

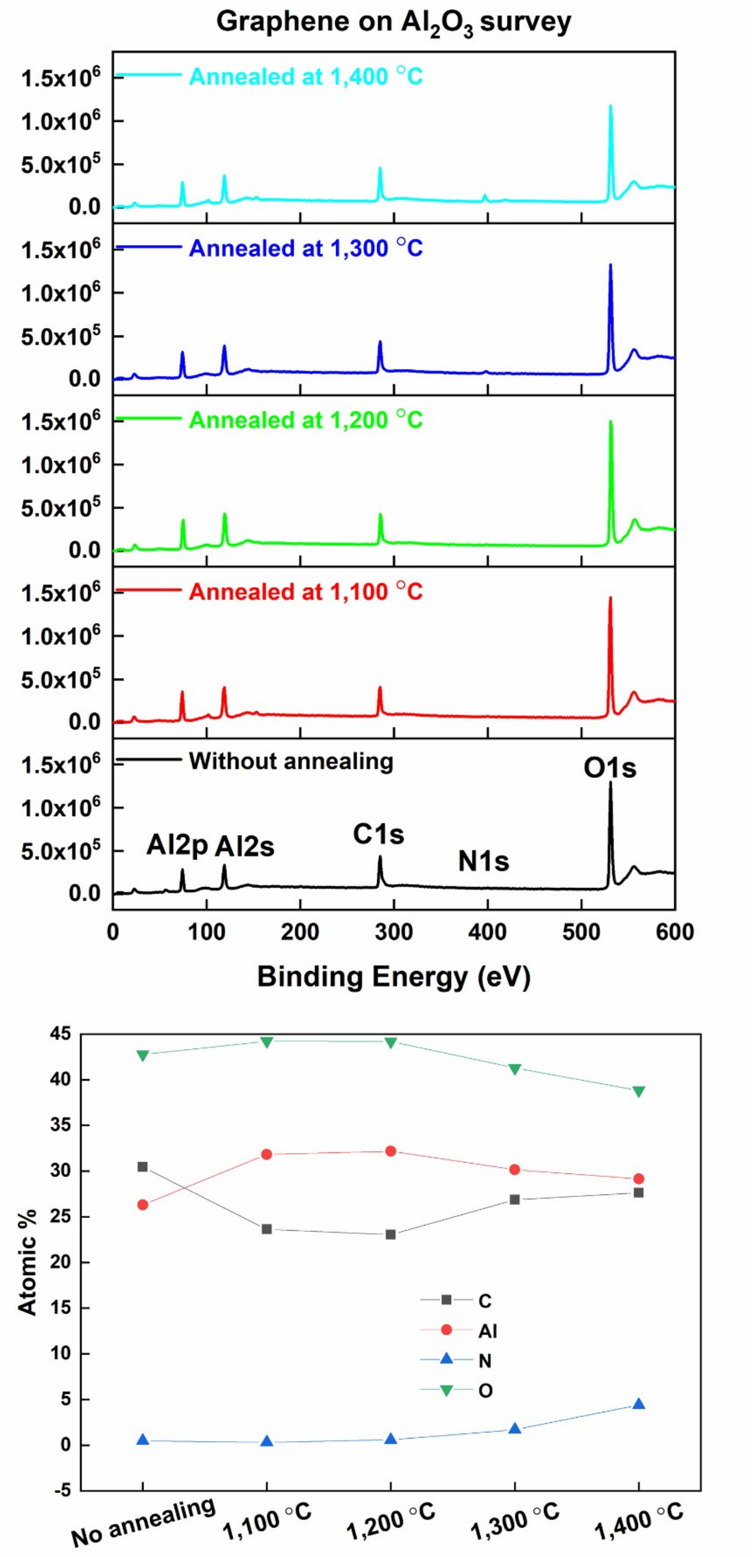

Figure. S9. XPS data for graphene on decomposed Al_2O_3 and its total atomic percentage. Although Al_2O_3 decomposed over 1,200 °C, the amplitude of C 1s did not considerably change compared with that in Figure 2a.

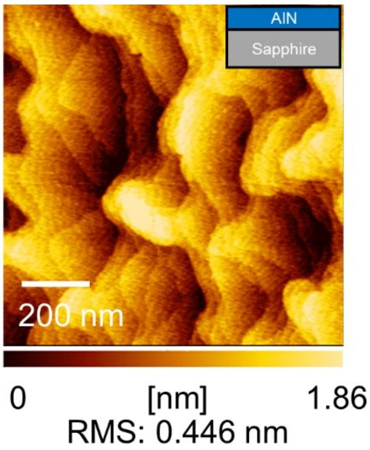

Figure. S10. Surface morphology of bare AlN layer fabricated by sputtering and high temperature annealing process.

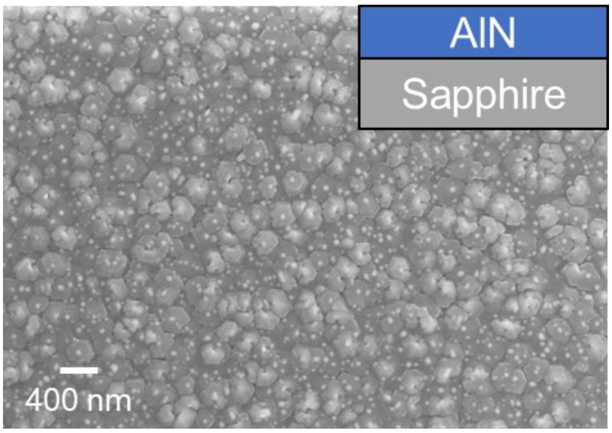

Figure. S11. Top-view SEM image of the grown AlN on *c*-plane Al_2O_3 at 1,380 °C

Reference

1 H. Miyake, C. -H. Lin, K. Tokoro, K. Hiramatsu, *J. Cryst. Growth* **2016**, *456,* 155-159.

2 X. Yang, S. Nitta, K. Nagamatsu, S. -Y. Bae, H. -J. Lee, Y. Liu, M. Pristovsek, Y. Honda, H. Amano, *J. Cryst. Growth* **2018**, *482,* 1-8.

3 X. Yang, S. Nitta, M. Pristovsek, Y. Liu, K. Nagamatsu, M. Kushimoto, Y. Honda, H. Amano, *Appl. Phys. Express* **2018**, *11*, 051002.

4 X. Yang, S. Nitta, M. Pristovsek, Y. Liu, Y. Liao, M. Kushimoto, Y. Honda, H. Amano, *2D Mater.* **2020**, *7*, 015004.

5 H. Amano, *Angew. Chem. Int. Ed.* **2015***, 54, 7764.*
